# TMEM200A is a potential prognostic biomarker and correlated with immune infiltrates in gastric cancer

**DOI:** 10.7717/peerj.15613

**Published:** 2023-06-29

**Authors:** Fujin Fang, Tiantian Zhang, Huan Lei, Xiaobing Shen

**Affiliations:** 1 Key Laboratory of Environmental Medical Engineering and Education Ministry, Southeast University, Nanjing, Jiangsu, China; 2 Department of Preventive Medicine, Southeast University, Nanjing, Jiangsu, China; 3 Department of Clinical Laboratory, The Third People’s Hospital of Bengbu, Bengbu, Anhui, China

**Keywords:** Gastric cancer, TMEM200A, TCGA, Prognosis, Immune infiltrates

## Abstract

**Background:**

Gastric cancer (GC) is one of the most common malignant tumors in the digestive system. Several transmembrane (TMEM) proteins are defined as tumor suppressors or oncogenes. However, the role and underlying mechanism of TMEM200A in GC remain unclear.

**Methods:**

We analyzed the expression of TMEM200A in GC. Furthermore, the influence of TMEM200A on survival of GC patients was evaluated. The correlations between the clinical information and TMEM200A expression were analyzed using chi-square test and logistic regression. Relevant prognostic factors were identified performing univariate and multivariate analysis. Gene set enrichment analysis (GSEA) was performed based on the TCGA dataset. Finally, we explore the relationship between TMEM200A expression and cancer immune infiltrates using CIBERSORT.

**Results:**

TMEM200A was up-regulated in GC tissues than that in adjacent non-tumor tissues based on TCGA database. Meta-analysis and RT-qPCR validated the difference in TMEM200A expression. Kaplan-Meier curves suggested the increased TMEM200A had a poor prognosis in GC patients. The chi-square test and logistic regression analyses showed that the TMEM200A expression correlates significantly with T stage. Multivariate analysis showed that TMEM200A expression might be an important independent predictor of poor overall survival in GC patients. GSEA identified five immune-related signaling pathways and five tumor-related signaling pathways significantly enriched in the high TMEM200A expression phenotype pathway. Finally, we found CD8+ T cells is apparently decreased in high TMEM200A expression group. Conversely, eosinophils is increased in high expression group compared with low expression group.

**Conclusion:**

TMEM200A is a potential prognostic biomarker and correlated with immune infiltrates in GC.

## Introduction

Gastric cancer (GC) is a deadly disease with low overall survival statistics worldwide. It ranks as the fifth most common human malignancy and the third leading cause of cancer-related deaths (Seidlitz, Koo & Stange, 2021). GC development is attributed to a variety of causes. *Helicobacter pylori* (*H. pylori*) infection is considered the major risk factor for non-cardiac GC (Smyth et al., 2020). Despite many strategies have been pursued to prevent GC, GC still remains a significant global health problem (Ferro et al., 2014; Rahman, Asombang & Ibdah, 2014). In light of cancer places a heavy burden on individuals, families, communities and health systems. New strategies are urgently needed to reveal the mechanism of the development and progression of GC. It is particularly important to identify new potential targets for GC therapy, as well as the identification of predictive biomarkers for clinical benefit.

Transmembrane (TMEM) proteins are a type of protein that span the lipid bilayer and mediate a large amount of biological functions (Jiang et al., 2021). Based on their transmembrane properties, TMEM proteins play an important role in signal transduction, ion transport and cell adhesion (Zhao et al., 2022). Some of them have been identified to be associated with diseases, including cancer (Ehrlich, Lacey & Ehrlich, 2020; Li et al., 2021a). In several cancer types, differential harmonization of gene expression of TMEMs have been found, such as breast cancer (TMEM17) (Zhao et al., 2018), lung cancer (TMEM116) (Zhang et al., 2021a), cervical cancer (TMEM48) (Jiang et al., 2021), gastric cancer (TMEM45B) (Shen et al., 2018), colorectal cancer (TMEM180) (Shiraishi et al., 2021), glioblastoma multiforme (TMEM39A) (Tran et al., 2017), hepatocellular carcinoma (TMEM106C) (Duan et al., 2021), osteosarcoma (TMEM45B) (Li et al., 2017).

The TMEM200s contain three TMEM200 family members in mammals, from TMEM200A to TMEM200C. Transmembrane protein 200A (TMEM200A), also known as KIAA1913, TTMA, and TTMC, is similar to other members of the gene family. In previous studies, researchers have demonstrated that TMEM200A is involved in the progression of pancreatic cancer (Tan, Schaffalitzky de Muckadell & Joergensen, 2020) and acute myeloid leukemia (AML) (Nie et al., 2022). In addition, evidence showed TMEM200A was important for adipose tissue morphology by regulation the proliferation of precursor cells. Both the accumulation of lipids and glycerol levels were reduced after knockdown of TMEM200A, while the cell number was increased (Lundback et al., 2020). However, TMEM200B has not been reported in cancer studies. Meanwhile, TMEM200C has been identified as a potential oncogene in uveal melanoma and is therefore a biomarker of early tumor metastasis and poor prognosis (Ness et al., 2021). In a previous, TMEM200A has demonstrated its oncogenic features in stomach adenocarcinoma (Zhang et al., 2020). However, the role of TMEM200A in GC is still unclear.

In this study, we compared the differential expression of TMEM200A expression in GC using publicly available data and experimental validation. Subsequently, we analyzed the influences of TMEM200A expression and patient characteristics on overall survival in GC patients. Additionally, we tried to unveil the underlying signaling pathway of TMEM200A expression level in GC progression. Moreover, we evaluated the association of TMEM200A with tumor-infiltrating immune cells. These, in turn will allow us to contribute towards the current literature on potential positive effects of TMEM200A in GC.

## Materials and Methods

### Data acquisition

The Cancer Genome Atlas program (TCGA), generated over 2.5 petabytes of genomic, epigenomic, transcriptomic, and proteomic data. We collected the RNA-seq data of GC patient and clinical information as previously described (Fang et al., 2022a). Patient characteristics are shown in Table S1.

A total of nine RNA-seq datasets were downloaded from another publicly available Gene Expression Omnibus (GEO) database, shown in Table S2. Data acquisition was the same as previously described (Chen et al., 2020).

### Expression analysis and survival analysis

RNA-seq data from TCGA were used to compare the expression of TMEM200A in normal and tumor tissues. Then, to verify the difference in TMEM200A expression in the TCGA database, a comprehensive meta-analysis of TMEM200A expression was conducted using GEO database. The methods were described in detail previously (Chen et al., 2020).

In order to study the influences of TMEM200A on overall survival. Firstly, we combined the mRNA expression with complete survival date. Shortly thereafter, tumor tissues were divided into two groups in accordance with the median expression of TMEM200A. At last, the Kaplan-Meier method was used to evaluate the prognosis of GC patients with differential expression of TMEM200A.

### Cell culture and clinical samples

Human gastric mucosal epithelial cells (GES-1) and human GC cell lines (HGC-27, MKN-28, AGS and MGC-803) were cultured in Roswell Park Memorial Institute (RPMI) 1640 (Gibco, Billings, MT, USA) or Dulbecco’s Modified Eagle Medium (DMEM) (Gibco, Billings, MT, USA), according to the specifications of the manufacturer’s datasheet and supplemented with 10% fetal bovine serum (FBS) at 37 °C in 5% CO_2_. All cell lines were purchased from Nanjing KeyGen Biotech Co., Ltd.

Thirty-five pairs of GC tissues and the adjacent non-tumor specimens were collected from Zhongda Hospital of Southeast University. All patients had never received preoperative radiotherapy or chemotherapy before surgery. The experiment was approved by the Ethics Committee of Zhongda Hospital of Southeast University (number: 2014ZDSYLL016.0), and all patients agreed and signed an informed consent form.

### RT-PCR and RT-qPCR

Total RNA was extracted using TRIzol (Invitrogen Life Technologies, Carlsbad, CA, USA), with a DNase digestion to remove genomic DNA contamination (GenStar). Reverse transcription was performed using RT-Phusion kit (Thermo Scientific, Waltham, MA, USA). Quantitative PCR was performed using the ΔΔCt method. The results were normalized to *β-actin*. The following primers were used: *β-actin*, TCCATCATGAAGTGTGACGT and GAGCAATGATCTTGATCTTCAT; TMEM200A, GCTGCCAGAAGACAGTTTGG and GGCACAAGCAACCTATCCAT.

### Univariate and multivariate Cox regression analyses

Univariate and multivariate Cox proportional hazard regression models were implemented to evaluate the influences of TMEM200A expression and patient characteristics on overall survival. The data were analyzed using R software.

### Gene set enrichment analysis (GSEA)

GSEA (version 3.0) was carried out to explore the significant survival differences between high expression and low expression of TMEM200A groups. Data analysis was based on TCGA database. We selected ‘c2.cp.kegg.v6.2.symbols.gmt’ as the reference gene set. The detailed parameter setting of the GSEA software was as described previously (Chen et al., 2020; Fang et al., 2022a).

### Gauging the immune response of 22 immune cells in GC by CIBERSORT

In this study, we gauged the immune response of 22 immune cells in GC *via* CIBERSORT, and thereby to assess its correlation with the expression of TMEM200A. We divided tumor samples into one half with high expression of TMEM200A and another half with low expression of TMEM200A using the median. The result is shown in a violin diagram.

### Statistical analysis

The differential expression of TMEM200A from TCGA was tested by Mann–Whitney U test. The data were analyzed using a Student’s *t* test for two-sample comparisons (*t* test was performed with equal variances) and a one-way analysis of variance (ANOVA) for multiple sample comparisons with Graphpad or SPSS software. In addition, the differential expression of TMEM200A from GEO database was tested by z-test. $\chi^{2}$ test and logistic regression were used to evaluate the interrelation between TMEM200A expression and patient characteristics. Multivariate Cox analysis was used to evaluate the influence of TMEM200A expression and other patient characteristics on survival. A *P*-value of <0.05 was considered significant.

## Results

### The differential expression of TMEM200A in GC

**Figure 1:**
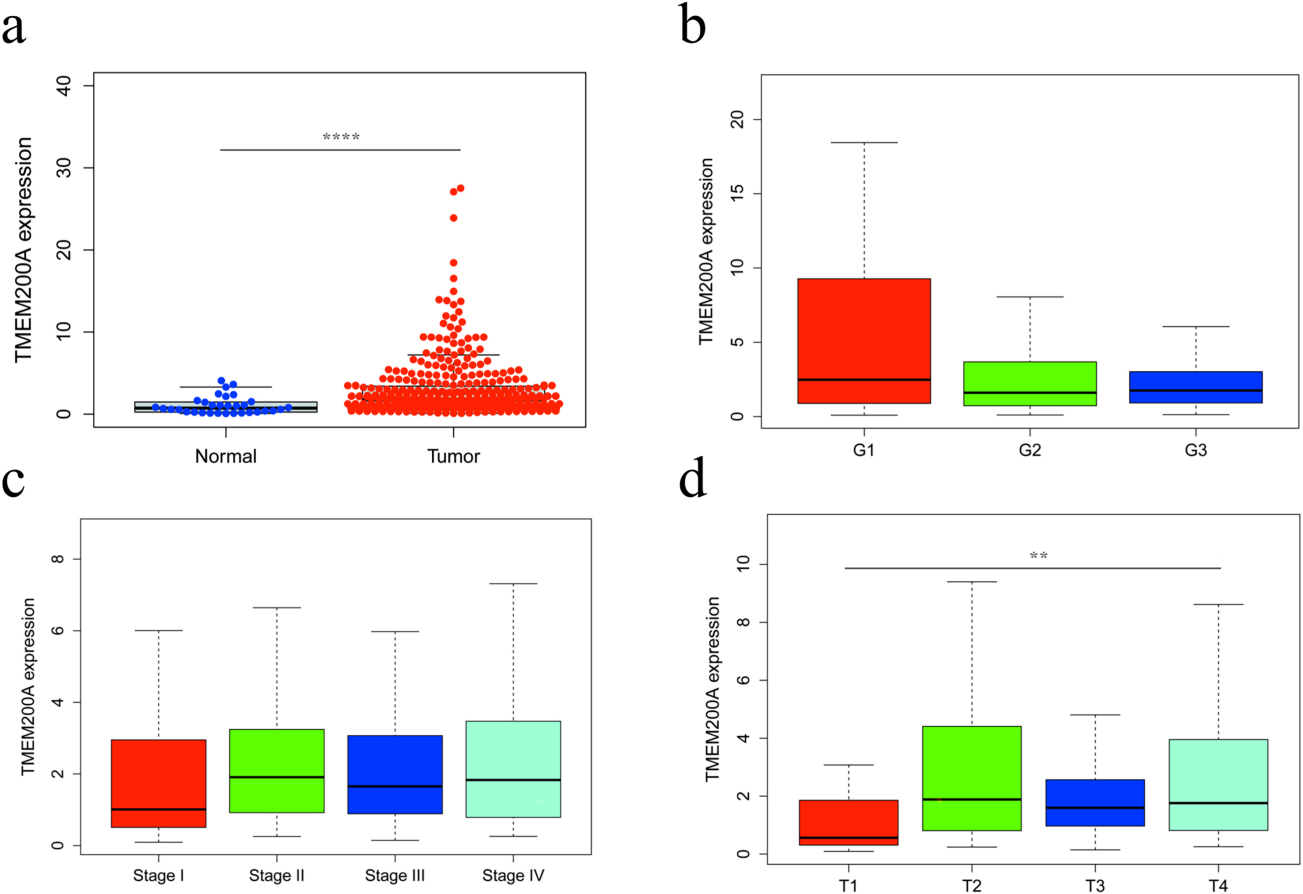
The differential expression of TMEM200A and its relationship with clinicopathological features based on TCGA data. (A) Differential expression of TMEM200A between GC tissues and adjacent non-tumor tissues. (B) The expression of TMEM200A is divided into groups by tumor differentiation (C), pathological stage (D) and T stage. **P < 0.001, ****P < 0.0001, unpaired two-sided Student’s *t*-test. Error bars indicate mean and SD. There are no statistically significant differences across tumor differentiation or pathological stage groups. Sample size: normal (*n* = 32), tumor (*n* = 375), G1 (*n* = 10), G2 (*n* = 137), G3 (*n* = 219), Stage I (*n* = 53), Stage II (*n* = 111), Stage III (*n* = 150), Stage IV (*n* = 38), T1 (*n* = 19), T2 (*n* = 80), T3 (*n* = 168), T4 (*n* = 100).

The GC mRNA dataset were downloaded from TCGA database. Our study represented a total of 407 samples with 375 samples of GC tissues and 32 samples of adjacent non-tumor tissues. We discovered that the expression of TMEM200A in GC tissues was significantly higher than that in adjacent non-tumor tissues (*P* = 1.382e−05) (Fig. 1A).

**Figure 2:**
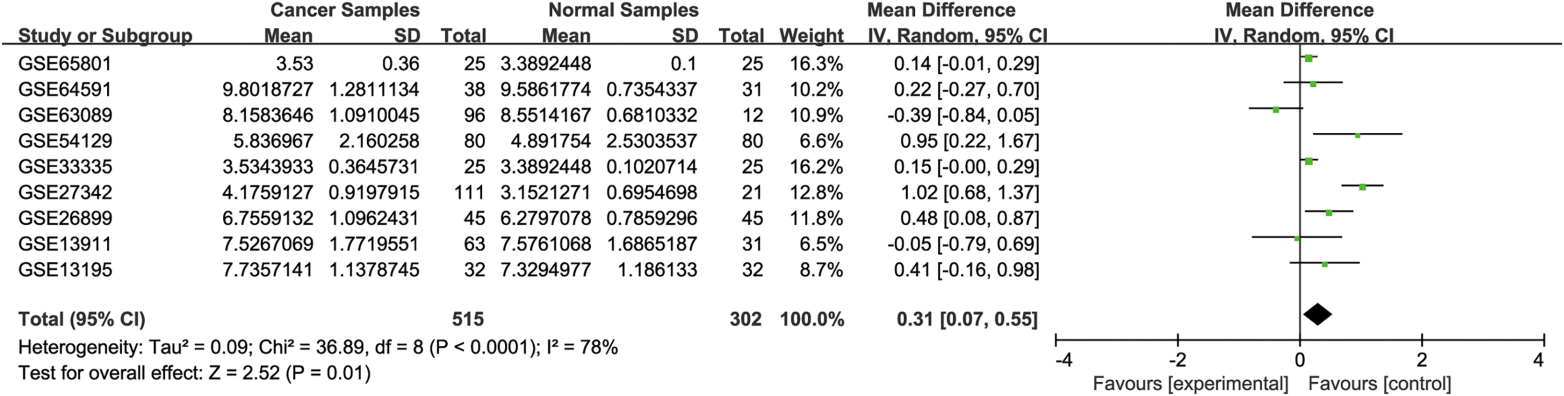
Forest plot of TMEM200A expression data from GEO Datasets. *I*^2^ = 78%, *P* = 0.01, z-test. The pooled SMD of TMEM200A is 0.31 (95% CI [0.07–0.55]) by the random effects model. SMD, standard mean difference; CI, confidence interval.

To validate the difference in TMEM200A expression in TCGA database, we performed a comprehensive meta-analysis of TMEM200A expression using GEO database. As a result, the *I*^2^ was 78% (*P* = 0.01), the combined SMD of TMEM200A was 0.31 according to the random effects model (95% CI [0.07–0.55], Fig. 2), suggesting that TMEM200A was up-regulated in GC.

**Figure 3:**
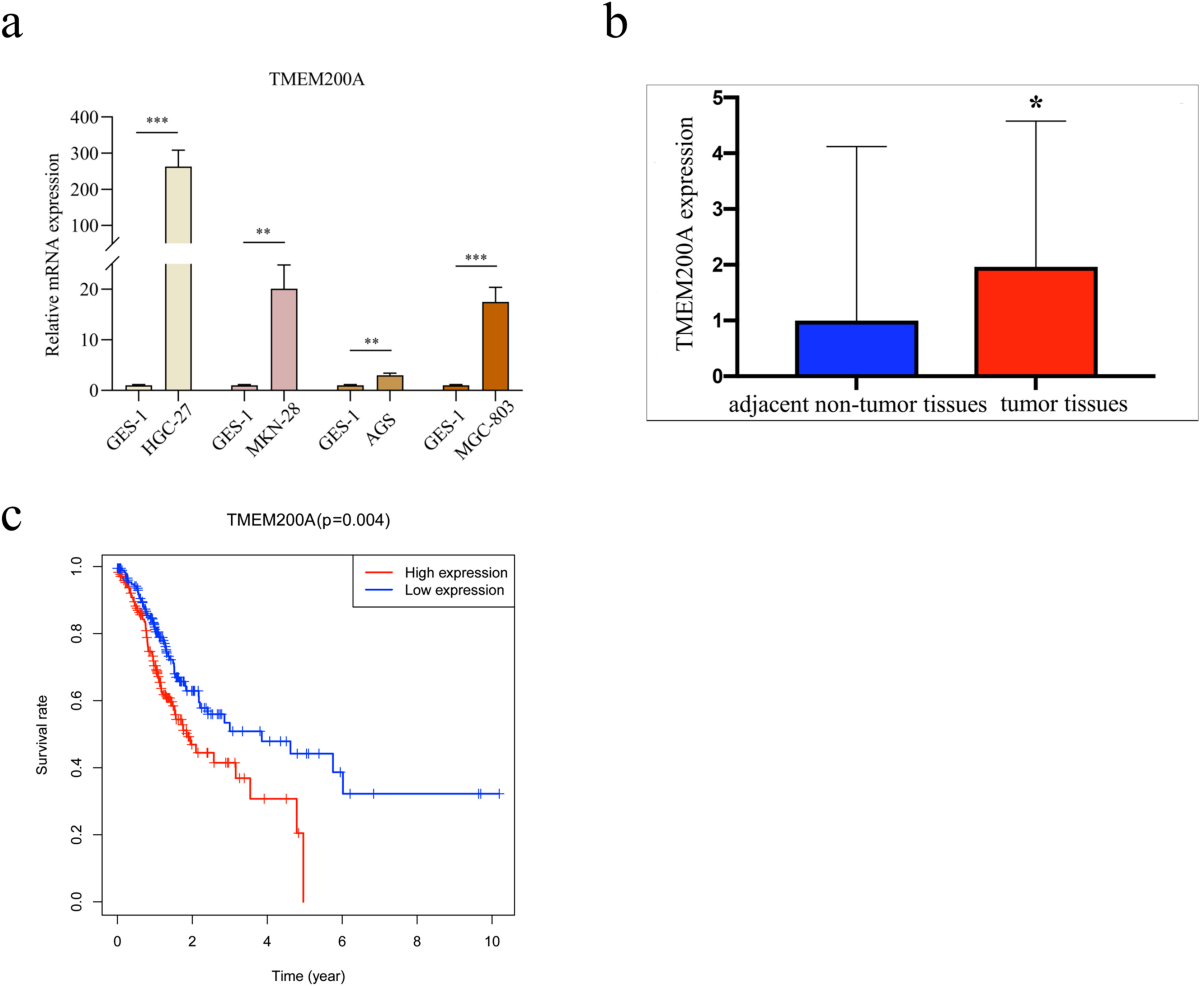
RT-qPCR analysis and survival analysis. (A) RT-qPCR analysis of TMEM200A mRNA expression in human gastric mucosal epithelial cells (GES-1) and human GC cell lines (HGC-27, MGC-803, AGS and MKN-28). **P < 0.01, ***P < 0.001, unpaired two sided Student’s *t* test. (B) RT-qPCR analysis of TMEM200A mRNA expression in clinical samples. (C) Kaplan-Meier curve for the relationship between TMEM200A expression and the prognosis of GC patients based on the TCGA database. *P < 0.05, unpaired two sided Student’s *t* test.

Finally, we experimentally verified the expression level of TMEM200A. As a result, the expression level of TMEM200A mRNA in GC cells (HGC-27, MKN-28, AGS and MGC-803) were significantly higher than that in GES-1 (*P* = 0.0006 for HGC-27 *vs*. GES-1; *P* = 0.0022 for MKN-28 *vs*. GES-1; *P* = 0.0019 for AGS *vs*. GES-1; *P* = 0.0006 for MGC-803 *vs*. GES-1) (Fig. 3A). In clinical samples, the expression level of TMEM200A was higher in tumor tissues compared with in adjacent non-tumor tissues (*P* = 0.018) (Fig. 3B).

### Survival analysis

Survival curve was performed to evaluate the prognosis of GC patients with differential expression of TMEM200A from TCGA database. The result showed that high expression of TMEM200A significantly corelated with poor prognosis of GC (*P* = 0.004) (Fig. 3C).

### TMEM200A expression and clinicopathological features

In order to explore the relationship between TMEM200A expression and clinicopathological features. Further analyses were conducted to analyze the expression level of TMEM200A in GC patients with patient characteristics. As a result, the expression level of TMEM200A was significantly different in group classified according to tumor T stage (*P* = 0.007) (Fig. 1D), while not in tumor differentiation (Fig. 1B) and pathological stage (Fig. 1C) groups. In addition, chi-square test showed that the up-regulated TMEM200A was significantly correlated with T stage (*P* = 0.002) (Table 1). Moreover, logistic regression analysis with TMEM200A expression also indicated that up-regulated TMEM200A was significantly related to age (OR = 1.29 for ≥65 *vs*. <65, *P* = 0.026) and T stage (OR = 3.99 for T2 *vs*. T1, *P* < 0.001; OR = 3.47 for T3 *vs*. T1, *P* < 0.001; OR = 4.19 for T4 *vs*. T1, *P* < 0.001) (Table 2).

**Table 1:** The relationship between TMEM200A expression and clinicopathological features in GC.

Clinicopathological features	TMEM200A expression		Total (*N*)	*P*-value
	High (*n* = 160)	Low (*n* = 159)		
Age				
<65 years	65 (49%)	69 (51%)	134	0.616
≥65 years	95 (51%)	90 (49%)	185	
Gender				
Male	94 (47%)	105 (53%)	199	0.179
Female	66 (55%)	54 (45%)	120	
Tumor differentiation				
G1 and G2	54 (46%)	63 (54%)	117	0.267
G3	106 (53%)	96 (47%)	202	
Pathological stage				
I–II	68 (48%)	74 (52%)	142	0.468
III–IV	92 (52%)	85 (48%)	177	
T classification				
T1–T2	71 (62%)	44 (38%)	115	0.002
T3–T4	89 (44%)	115 (56%)	204	
Lymph node metastasis				
Negative	51 (51%)	50 (49%)	101	0.934
Positive	109 (50%)	109 (50%)	218	
Distant metastasis				
No	149 (51%)	148 (49%)	297	0.988
Yes	11 (50%)	11 (50%)	22	

**Table 2:** TMEM200A expression correlated with clinicopathological features in GC.

Clinicopathological features	Total (*N*)	Odds ratio in TMEM200A expression	*P*-value
Age			
≥65 *vs*. <65	319	1.29 (1.03–1.61)	0.026
Gender			
Male *vs*. female	319	0.85 (0.67–1.06)	0.153
Tumor differentiation			
G2 *vs*. G1	117	0.84 (0.39–1.80)	0.657
G3 *vs*. G1	209	0.86 (0.40–1.83)	0.695
Pathological stage			
Stage II *vs*. stage I	145	1.16 (0.65–2.06)	0.618
Stage III *vs*. stage I	183	0.93 (0.44–1.98)	0.850
Stage IV *vs*. stage I	79	1.28 (0.50–3.23)	0.606
T classification			
T2 *vs*. T1	80	3.99 (2.19–7.26)	0.000
T3 *vs*. T1	168	3.47 (1.67–7.21)	0.000
T4 *vs*. T1	103	4.19 (1.94–9.05)	0.000
Lymph node metastasis			
Positive *vs*. negative	319	1.08 (0.73–1.58)	0.709
Distant metastasis			
Yes *vs*. no	319	0.68 (0.34–1.35)	0.270

### Prognostic significance of TMEM200A expression in GC patients

**Figure 4:**
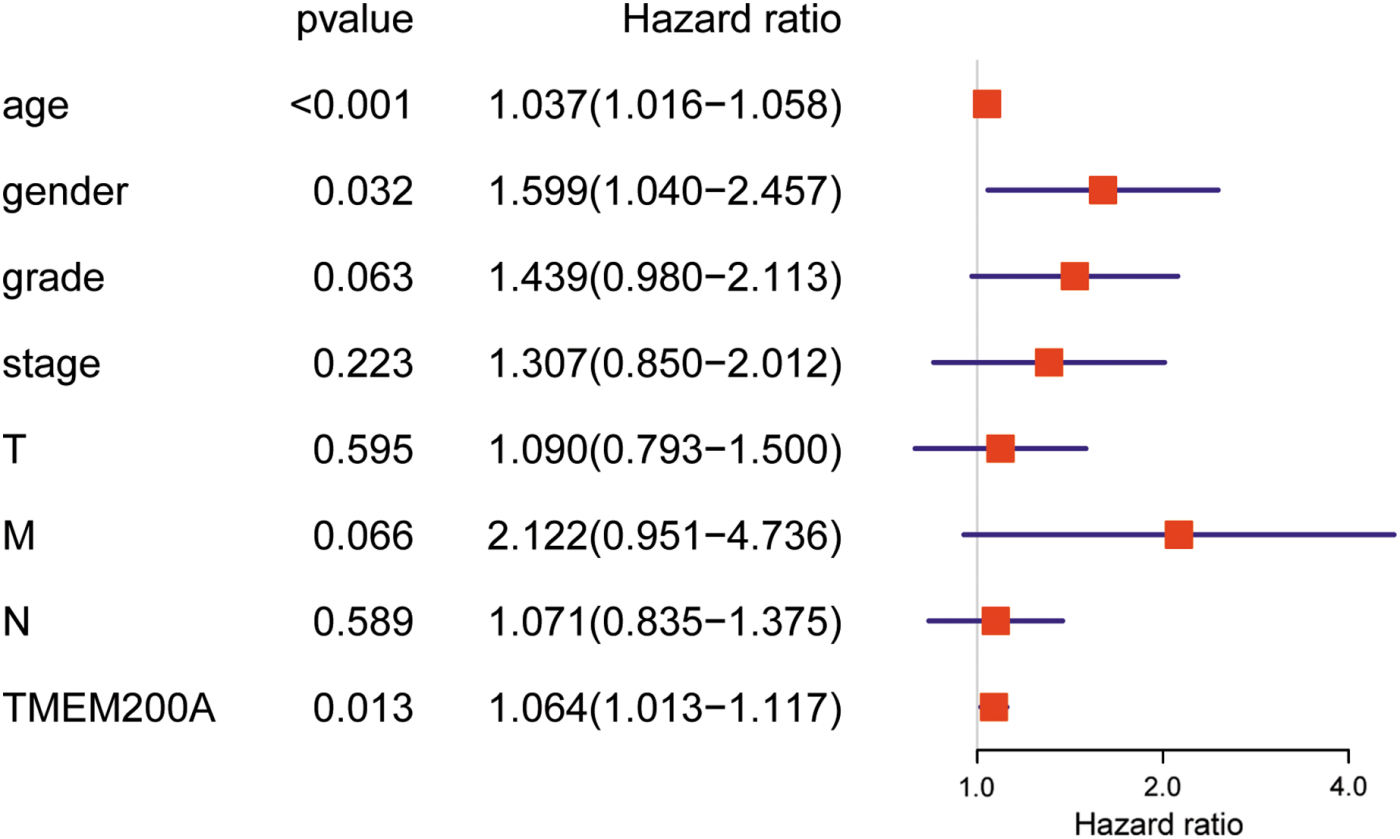
Forest plot for the multivariate Cox proportional hazard regression model. TMEM200A could act as an independent predictor of poor overall survival rate (HR, 1.064; 95% CI [1.013–1.117]; *P* = 0.013) in GC patients. HR, hazard ratio; CI, confidence interval.

Previous analysis suggested that the overall survival was significantly correlated with TMEM200A expression in GC patients. Here, univariate and multivariate analysis were conducted to assess the effect of TMEM200A expression and patient characteristics on overall survival. Univariate analysis revealed that TMEM200A (HR = 1.064; 95% CI [1.018–1.113]; *P* = 0.006), age (HR = 1.027; 95% CI [1.008–1.047]; *P* = 0.006), pathological stage (HR = 1.535; 95% CI [1.221–1.931]; *P* = 0.000), T stage (HR = 1.298; 95% CI [1.023–1.645]; *P* = 0.032), M stage (HR = 2.048; 95% CI [1.096–3.827]; *P* = 0.025) and N stage (HR = 1.267; 95% CI [1.069–1.502]; *P* = 0.006), which were significant predictors of poor prognosis (Table 3). Multivariate analysis showed that TMEM200A (HR = 1.064; 95% CI [1.013–1.117]; *P* = 0.013), age (HR = 1.037; 95% CI [1.016–1.058]; *P* = 0.000) and gender (HR = 1.599; 95% CI [1.041–2.457]; *P* = 0.032) were independently associated with overall survival in GC (Table 3) (Fig. 4). These results suggested TMEM200A is a potential freestanding predictor of poor overall survival in GC patients.

**Table 3:** Univariate and multivariate analysis of the relationship between TMEM200A expression and GC patients.

Clinicopathological features	Univariate analysis			Multivariate analysis		
	HR	95% CI	*P*-value	HR	95% CI	*P*-value
Age	1.027	[1.008–1.047]	0.006	1.037	[1.016–1.058]	0.000
Gender	1.484	[0.980–2.247]	0.062	1.599	[1.041–2.457]	0.032
Grade	1.368	[0.947–1.977]	0.095	1.439	[0.980–2.113]	0.063
Pathological stage	1.535	[1.221–1.931]	0.000	1.308	[0.850–2.012]	0.222
T	1.298	[1.023–1.645]	0.032	1.090	[0.793–1.500]	0.595
M	2.048	[1.096–3.827]	0.025	2.122	[0.951–4.736]	0.066
N	1.267	[1.069–1.502]	0.006	1.071	[0.835–1.375]	0.589
TMEM200A	1.064	[1.018–1.113]	0.006	1.064	[1.013–1.117]	0.013

**Note:**

HR, hazard ratio; CI, confidence interval.

### Gene sets enriched in TMEM200A expression phenotype

**Figure 5:**
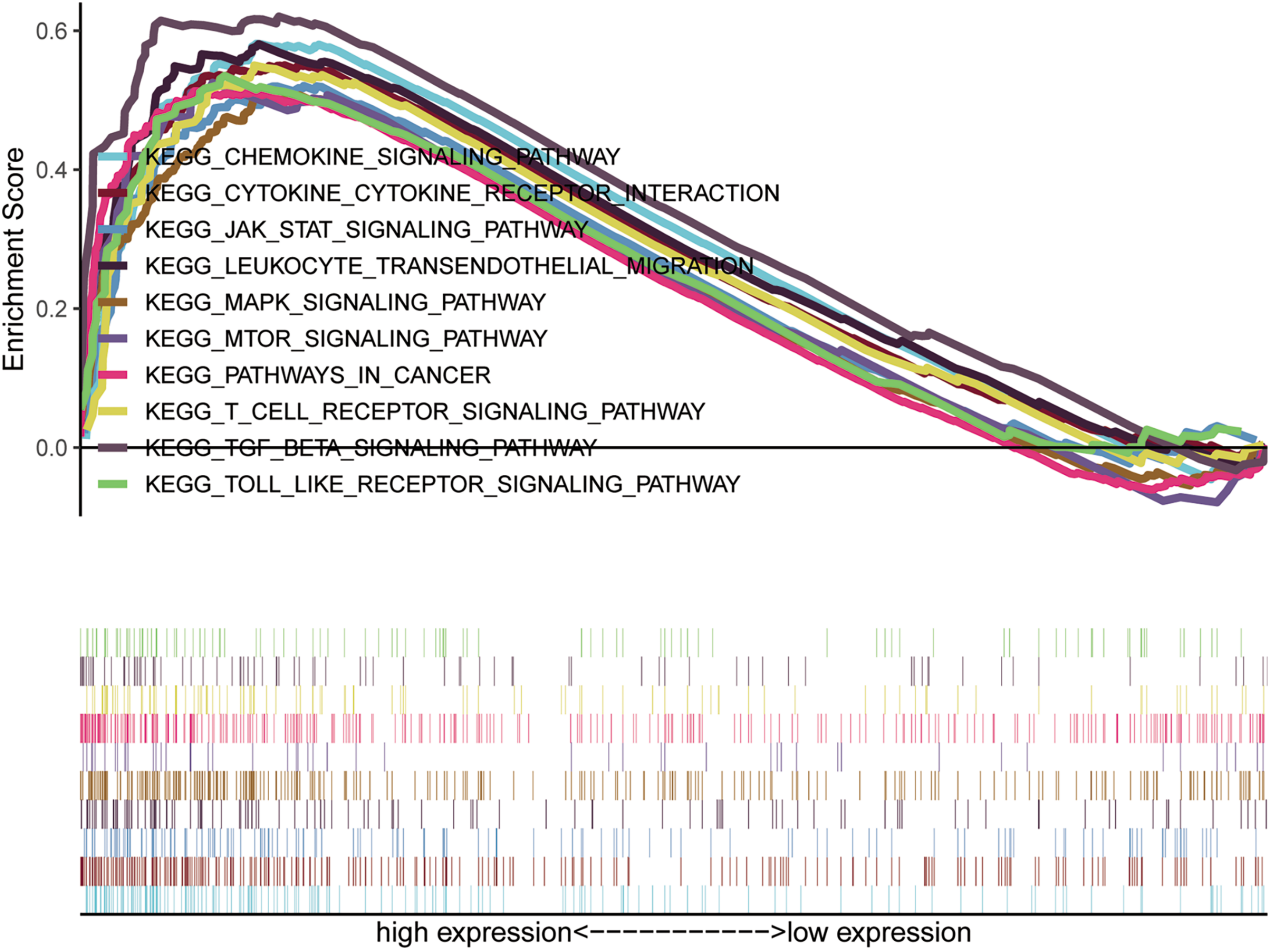
A combined enrichment maps from genomic enrichment analysis. Gene Set Enrichment Analysis (GSEA) results showing differential enrichment of genes in KEGG with high TEME200A expression.

High expression TMEM200A related signaling pathways based on GSEA was used to identify signaling pathways involved in GC. Based on normalized enrichment score (NES), false discovery rate (FDR) *q*-value, and nominal *P*-value, significantly enriched signaling pathways were obtained. 10 KEGG items including cytokine-cytokine receptor interaction, chemokine signaling pathway, T cell receptor signaling pathway, leukocyte transendothelial migration, toll-like receptor signaling pathway, TGF-β signaling pathway, JAK-STAT signaling pathway, mTOR signaling pathway, MAPK signaling pathway, pathway in cancer were significantly enriched in the increased expression phenotypes of TMEM200A (Table 4) (Figs. 5 and S1).

**Table 4:** Gene sets enriched in the high-TMEM200A expression phenotype.

TMEM200A expression level	Gene set name	NES	NOM *P*-value	FDR *q*-value
High-TMEM200A expression	KEGG_CYTOKINE_CYTOKINE_RECEPTOR_INTERACTION	1.99	0.004	0.018
	KEGG_CHEMOKINE_SIGNALING_PATHWAY	1.96	0.004	0.020
	KEGG_T_CELL_RECEPTOR_SIGNALING_PATHWAY	1.72	0.030	0.052
	KEGG_LEUKOCYTE_TRANSENDOTHELIAL_MIGRATION	1.95	0.004	0.020
	KEGG_TOLL_LIKE_RECEPTOR_SIGNALING_PATHWAY	1.80	0.016	0.042
	KEGG_TGF_BETA_SIGNALING_PATHWAY	2.09	0.000	0.010
	KEGG_JAK_STAT_SIGNALING_PATHWAY	1.91	0.004	0.023
	KEGG_MTOR_SIGNALING_PATHWAY	1.71	0.022	0.052
	KEGG_MAPK_SIGNALING_PATHWAY	1.96	0.000	0.018
	KEGG_PATHWAYS_IN_CANCER	1.90	0.000	0.022

**Note:**

NES, normalized enrichment score; NOM, nominal; FDR, false discovery rate.

### Relationship between TMEM200A expression and tumor-infiltrating immune cells

**Figure 6:**
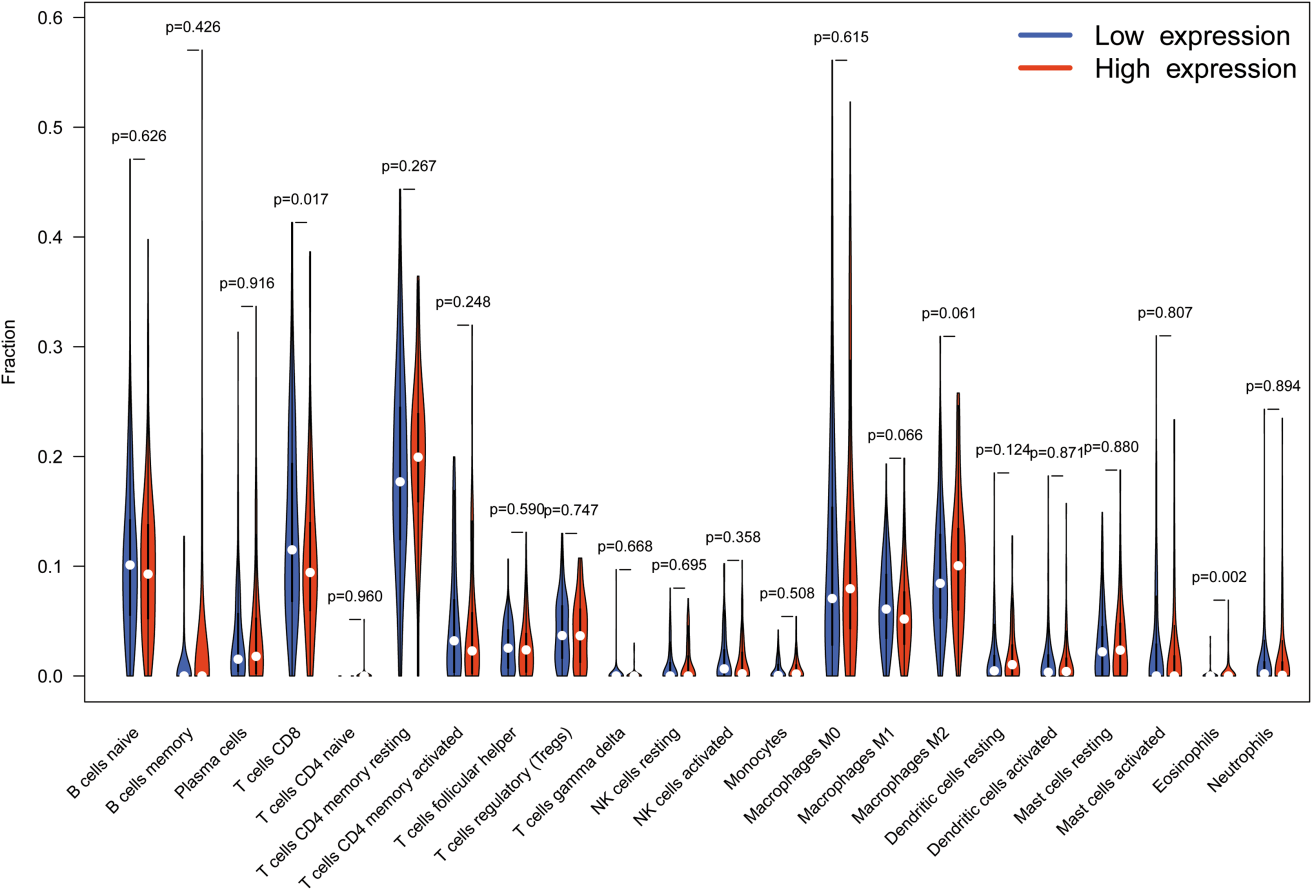
The proportion of 22 subpopulations of immune cells (T cells CD8 and Eosinophils are main immune cells effected by TMEM200A expression). Among them, T cells CD8 (*P* = 0.017) is apparently decreased in high expression group compared with low expression group. In contrast, eosinophils (*P* = 0.002) is increased in high expression group compared with low expression group.

Gene set enrichment analysis suggest several immune-related signaling pathway as high expression TMEM200A associated signaling pathways in GC. Here, we next investigated whether TMEM200A expression was correlated to immune infiltration in GC. 375 tumor samples were divided into two parts according to TMEM200A expression, and thereby 187 samples of high expression group and 188 samples of low expression group met screening criterion. Finally, CIBERSORT was used to explore gene expression profiles of downloaded samples to infer the density of 22 types of immune cells, which helped assess their differing concentrations in the up-regulated and down-regulated TMEM200A expression groups. The proportion of 22 subpopulations of immune cells were showed in Fig. 6. CD8+ T cells and eosinophils are main immune cells effected by TMEM200A expression. Among them, CD8+ T cells are apparently (*P* = 0.017) decreased in high expression group compared with low expression group. In contrast, eosinophils (*P* = 0.002) are increased in high expression group compared with low expression group.

## Discussion

TMEMs make up approximately 30% of the human proteome (Babcock & Li, 2014). Most of them play a fundamental role during tumor progression or cancer cell metastasis *via* signal transduction (Marx et al., 2020). In previous studies, multiple mechanisms indicated TMEMs are involved in tumor progression, as examples some TMEMs have been described as tumor suppressors. The overexpression of TMEM176A can inhibit cell migration, invasion and cell growth in colorectal (Gao et al., 2017). Furthermore, the expression levels of both TMEM97 protein and mRNA were lower in tumor tissues compared to adjacent normal tissues in pancreatic cancer and kidney cancer (Kayed et al., 2004; Schmit & Michiels, 2018). In the opposite site, many TMEMs are up-regulated in cancer, thereby TMEMs can act as oncogenes. In a previous study, it has been evidenced that TMEM158 can promote tumor growth, such as regulating cell proliferation and invasion in ovarian cancer (Cheng et al., 2015). In addition, inhibition of TMEM158 can impair the TGF-β signaling pathway (Cheng et al., 2015). Additionally, many TMEMs have been involved in drug resistance, such as TMEM45A (Flamant et al., 2012). Evidence showed that high levels of TMEM45A expression in breast and liver cancer cells may be indicative of potential resistance to cancer therapy, making TMEM45A a tumor-promoting factor (Flamant et al., 2012). Thus, TMEM can function either as an oncoprotein or tumor suppressor. Nevertheless, the reporting of TMEM200A was extremely inadequate, which hampers the assessment of its role in tumor progression. Here we have used publicly available data, RT-qPCR, survival curve and Cox proportional hazard regression models to gain new insights into the expression of TMEM200A on the overall survival in GC patients. In addition, the relationship between TMEM200A expression and tumor-infiltrating immune cells were also analyzed. These insights may have impact on GC patient care.

Here, we found that the differential expression of TMEM200A was significantly up-regulated in GC tissues than that in adjacent normal tissues in accordance to TCGA database. Consequentially, a meta-analysis was performed to verify the differential expression of TMEM200A in GC based on GEO datasets. Consistent with the previous observations, TMEM200A was increased in GC tissues. Furthermore, it was also confirmed in our experiments with GES-1 and GC cells in addition to human GC tissues and adjacent normal tissues, and the result was consistent with the results of the bioinformatics assay. Moreover, we analyzed the expression level of TMEM200A in GC patients with patient characteristics. As a result, the expression level of TMEM200A was significantly different in group classified according to tumor T stage. In addition, chi-square test and logistic regression analysis with TMEM200A expression also indicated that up-regulated TMEM200A was significantly related to T stage. Collectively, these results suggested that the high expression of TMEM200A may act as an oncogene, and thereby can be a prognostic biomarker in GC.

Our study uncovered the relationship between TMEM200A expression with the overall survival of GC patients using Kaplan-Meier method. We showed that GC patients with high TMEM200A expression had a poorer overall survival than that of patients with low expression. In addition, univariate analysis showed that TMEM200A and some other patient characteristics, as examples age, pathological stage, T stage, M stage and N stage were poor prognosis predictor. Moreover, multivariate analysis showed that TMEM200A was independently associated with overall survival in GC. These findings suggested TMEM200A is a freestanding predictor of poor overall survival in GC patients.

It has been demonstrated that TMEMs were involved in cancer progression *via* different signaling pathways. TMEM116, for example, is required for lung cancer cell motility and metastasis through PDK1 signaling pathway (Zhang et al., 2021a); TMEM229A suppresses non‑small cell lung cancer progression *via* inactivating the ERK pathway (Zhang et al., 2021b); inhibition of proliferation by knockdown of TMEM168 in glioblastoma cells *via* suppression of Wnt/β-Catenin pathway (Xu et al., 2019); TMEM17 promotes malignant progression of breast cancer *via* AKT/GSK3β signaling (Zhao et al., 2018). In this situation, 10 KEGG items including cytokine-cytokine receptor interaction, chemokine signaling pathway, T cell receptor signaling pathway, leukocyte transendothelial migration, Toll-like receptor signaling pathway, TGF-β signaling pathway, JAK-STAT signaling pathway, mTOR signaling pathway, MAPK signaling pathway, pathway in cancer were significantly enriched in the increased expression phenotypes of TMEM200A using GSEA. The cytokine-cytokine receptor interaction is an immune-related signaling pathway and thereby indicated that TMEM200A may regulate the inflammatory response and immune function in GC (Qian, Zhang & Wang, 2019; Song et al., 2020). The chemokine signaling pathway, which may contribute to chronic inflammation (Zhang et al., 2015), as several cancers were found to be associated with chronic inflammatory conditions (Malhab et al., 2021), including GC (Sammarco et al., 2019). T cell receptor signaling pathway has a central role in the control of T cell differentiation, homeostasis and function, such as T cell receptor signaling play an important role in the differentiation and function of Treg cell (Li & Rudensky, 2016), and thus suppress host immunity (Fang et al., 2022b). Leukocyte transendothelial migration is one of the most step in the initiation of inflammatory immune response and chronic inflammation can lead to destructive diseases. Leukocyte migration inhibitors are considered as promising and potentially effective therapeutic agents to treat inflammatory (Getter et al., 2019; Kong et al., 2018). Toll-like receptor signaling pathway has been established to play an essential role in the activation of innate immunity and is one potential common inflammatory pathway (Figueroa-Hall, Paulus & Savitz, 2020; Takeda & Akira, 2004). Dysregulation of this signaling pathway can result in development of cancer (Moradi-Marjaneh et al., 2018). JAK-STAT signaling pathway is involved in many crucial biological processes, including cell proliferation, differentiation, apoptosis, and immune regulation (Xin et al., 2020). MAPK signaling pathway plays an important role in various biological events, including in the differentiation, proliferation, apoptosis of cells and metabolic reprogramming (Asl et al., 2021). TGF-β signaling pathway (Colak & ten Dijke, 2017; Xiong et al., 2020), mTOR signaling pathway (Fattahi et al., 2020; Zhang et al., 2019) and pathway in cancer (Chen et al., 2020; Li et al., 2021b) are common tumor-associated signaling pathways, including in GC. Taken together, the above findings not only provide ideas to explore the carcinogenic and cancer-promoting molecular mechanisms of TMEM200A, but also indicate TMEM200A is correlated with immune infiltrates in GC, suggesting that TMEM200A is involved in GC progression *via* regulating various molecular signaling pathways.

As TMEM200A is involved in several immune-associated signaling pathways. Here, our studies revealed that in GC diverse immune infiltration levels are correlated with TMEM200A expression. A CIBERSORT analysis showed a negative connection of TMEM200A expression with infiltration level of CD8+ T cells. CD8+ T cells are considered the main effectors of anti-tumor immunity in tumor microenvironment (Hossain et al., 2021). Thereafter, decreased level of CD8+ T cells or CD8+ T cells exhaustion can lead to a bad prognosis (Dolina et al., 2021; Fang et al., 2022b; Kurachi, 2019). In contrast, TMEM200A had a positive connection with infiltration level of eosinophils, where, tumor-associated tissue eosinophils appear to be a good prognostic factor in gastrointestinal (Reichman, Karo-Atar & Munitz, 2016). Thus, the relationships between gene markers of immune cells and TMEM200A expression implicate the significant meaning of TMEM200A in regulating tumor immune microenvironment of GC.

Striking, there is a recent publication that has similar methodology and results to ours (Deng et al., 2023). In this study, the authors also found the expression of TMEM200 in GC tissues was significantly higher than that in normal tissues. Subsequently, survival curve and Cox proportional hazard regression models indicated that high expression of TMEM200A was associated with a poor prognosis in GC patients. In addition, the possible biological functions and signaling pathways involved in TMEM200A and the relationship between TMEM200A expression and tumor-infiltrating immune cells were also analyzed. Moreover, co-expression of genes with TMEM200A, the correlation between TMEM200A expression and immune checkpoint expression, the effect of TMEM200A on the sensitivity of common chemotherapeutic drugs and DNA methylation sites were also analyzed in this study. However, the limitation is that this research work is based entirely on public databases. In our study, in order to reduce bias, we used multiple GEO datasets to validate the differential expression of TMEM200A. Furthermore, chi-square test and logistic regression were used to analysis the expression level of TMEM200A in GC patients with patient characteristics. Additionally, we experimentally validated the differential expression of TMEM200A in cell lines and clinical tissues. To sum up, the two studies yielded similar results and shed light on the potential role of TMEM200A in GC.

## Conclusions

In summary, our study points to TMEM200A as a potential prognostic biomarker and correlated with immune infiltrates in GC. However, the prognostic value of TMEM200A in GC still needs to be explored and validated. For better understand the fundamental role of TMEM200A in GC, large amount in-depth *in vivo* and *in vitro* research is urgently needed to reveal the mechanism of TMEM200A in the development and progression of GC. We look forward to providing more substantial benefits in addressing critical issues.

## Supplemental Information

10.7717/peerj.15613/supp-1 Supplemental Information 1 The significantly enriched signaling pathways associated with the increased TMEM200A expression. (a) Cytokine-cytokine receptor interaction, (b) chemokine signaling pathway, (c) T cell receptor signaling pathway, (d) leukocyte transendothelial migration, (e) Toll-like receptor signaling pathway, (f) TGF-β signaling pathway, (g) JAK-STAT signaling pathway, (h) mTOR signaling pathway, (i) MAPK signaling pathway, (j) pathway in cancer.

10.7717/peerj.15613/supp-2 Supplemental Information 2 Clinicopathological features of patients with GC.

10.7717/peerj.15613/supp-3 Supplemental Information 3 Relevant information of the selected GEO series dataset.

## Additional Information and Declarations

### Data Availability

The following information was supplied regarding data availability: The gastric cancer patient RNA-seq data, and clinical information were downloaded from TCGA (https://portal.gdc.cancer.gov/), and included 32 adjacent non-tumor tissues and 375 tumor tissues: TMEM200A. The RNA-seq datasets are available at Gene Expression Omnibus (GEO) database: GSE13195, GSE13911, GSE26899, GSE27342, GSE33335, GSE54129, GSE63089, GSE64591, GSE65801. The raw data for Figures 3 & 6 and Tables 1–4 are available at Figshare: Fang, Fujin (2023): Raw data for Peer J.rar. figshare. Dataset. https://doi.org/10.6084/m9.figshare.22032611.v1.

## References

[ref-1] Asl ER, Amini M, Najafi S, Mansoori B, Mokhtarzadeh A, Mohammadi A, Lotfinejad P, Bagheri M, Shirjang S, Lotfi Z, Rasmi Y, Baradaran B (2021). Interplay between MAPK/ERK signaling pathway and MicroRNAs: a crucial mechanism regulating cancer cell metabolism and tumor progression. Life Sciences.

[ref-2] Babcock JJ, Li M (2014). Deorphanizing the human transmembrane genome: A landscape of uncharacterized membrane proteins. Acta Pharmacologica Sinica.

[ref-3] Chen X, Li X, Hu X, Jiang F, Shen Y, Xu R, Wu L, Wei P, Shen X (2020). LUM expression and its prognostic significance in gastric cancer. Frontiers in Oncology.

[ref-4] Cheng Z, Guo J, Chen L, Luo N, Yang W, Qu X (2015). Overexpression of TMEM158 contributes to ovarian carcinogenesis. Journal of Experimental & Clinical Cancer Research.

[ref-5] Colak S, ten Dijke P (2017). Targeting TGF-beta signaling in cancer. Trends in Cancer.

[ref-6] Deng HY, Li TF, Wei FX, Han W, Xu XD, Zhang YC (2023). High expression of TMEM200A is associated with a poor prognosis and immune infiltration in gastric cancer. Pathology and Oncology Research.

[ref-7] Dolina JS, Van Braeckel-Budimir N, Thomas GD, Salek-Ardakani S (2021). CD8(+) T cell exhaustion in cancer. Frontiers in Immunology.

[ref-8] Duan J, Qian Y, Fu X, Chen M, Liu K, Liu H, Yang J, Liu C, Chang Y (2021). TMEM106C contributes to the malignant characteristics and poor prognosis of hepatocellular carcinoma. Aging.

[ref-9] Ehrlich KC, Lacey M, Ehrlich M (2020). Epigenetics of skeletal muscle-associated genes in the ASB, LRRC, TMEM, and OSBPL gene families. Epigenomes.

[ref-10] Fang F, Liu C, Li Q, Xu R, Zhang T, Shen X (2022a). The role of SETBP1 in gastric cancer: friend or foe. Frontiers in Oncology.

[ref-11] Fang F, Zhang T, Li Q, Chen X, Jiang F, Shen X (2022b). The tumor immune-microenvironment in gastric cancer. Tumori Journal.

[ref-12] Fattahi S, Amjadi-Moheb F, Tabaripour R, Ashrafi GH, Akhavan-Niaki H (2020). PI3K/AKT/mTOR signaling in gastric cancer: epigenetics and beyond. Life Sciences.

[ref-13] Ferro A, Peleteiro B, Malvezzi M, Bosetti C, Bertuccio P, Levi F, Negri E, La Vecchia C, Lunet N (2014). Worldwide trends in gastric cancer mortality (1980–2011), with predictions to 2015, and incidence by subtype. European Journal of Cancer.

[ref-14] Figueroa-Hall LK, Paulus MP, Savitz J (2020). Toll-like receptor signaling in depression. Psychoneuroendocrinology.

[ref-15] Flamant L, Roegiers E, Pierre M, Hayez A, Sterpin C, De Backer O, Arnould T, Poumay Y, Michiels C (2012). TMEM45A is essential for hypoxia-induced chemoresistance in breast and liver cancer cells. BMC Cancer.

[ref-16] Gao D, Han YJ, Yang Y, Herman JG, Linghu EQ, Zhan QM, Fuks F, Lu ZJ, Guo MZ (2017). Methylation of TMEM176A is an independent prognostic marker and is involved in human colorectal cancer development. Epigenetics.

[ref-17] Getter T, Margalit R, Kahremany S, Levy L, Blum E, Khazanov N, Keshet-Levy NY, Tamir TY, Ben Major M, Lahav R, Zilber S, Senderowitz H, Bradfield P, Imhof BA, Alpert E, Gruzman A (2019). Novel inhibitors of leukocyte transendothelial migration. Bioorganic Chemistry.

[ref-18] Hossain MA, Liu G, Dai B, Si Y, Yang Q, Wazir J, Birnbaumer L, Yang Y (2021). Reinvigorating exhausted CD8(+) cytotoxic T lymphocytes in the tumor microenvironment and current strategies in cancer immunotherapy. Medicinal Research Reviews.

[ref-19] Jiang XY, Wang L, Liu ZY, Song WX, Zhou M, Xi L (2021). TMEM48 promotes cell proliferation and invasion in cervical cancer via activation of the Wnt/beta-catenin pathway. Journal of Receptors and Signal Transduction.

[ref-20] Kayed H, Kleeff J, Ding J, Hammer J, Giese T, Zentgraf H, Buchler MW, Friess H (2004). Expression analysis of MAC30 in human pancreatic cancer and tumors of the gastrointestinal tract. Histology and Histopathology.

[ref-21] Kong DH, Kim YK, Kim MR, Jang JH, Lee S (2018). Emerging roles of vascular cell adhesion molecule-1 (VCAM-1) in immunological disorders and cancer. International Journal of Molecular Sciences.

[ref-22] Kurachi M (2019). CD8(+) T cell exhaustion. Seminars in Immunopathology.

[ref-23] Li X, Chen X, Hu X, Shen Y, Xu R, Wu L, Shen X (2021b). Overexpression of GUCY1A2 correlates with poor prognosis in gastric cancer patients. Frontiers in Oncology.

[ref-24] Li Y, Guo W, Liu S, Zhang B, Yu BB, Yang B, Kan SL, Feng SQ (2017). Silencing transmembrane protein 45B (TNEM45B) inhibits proliferation, invasion, and tumorigenesis in osteosarcoma cells. Oncology Research Featuring Preclinical and Clinical Cancer Therapeutics.

[ref-25] Li C, Ou R, Chen Y, Gu X, Wei Q, Cao B, Zhang L, Hou Y, Liu K, Chen X, Song W, Zhao B, Wu Y, Liu Y, Shang H (2021a). Mutation analysis of TMEM family members for early-onset Parkinson’s disease in Chinese population. Neurobiology of Aging.

[ref-26] Li MO, Rudensky AY (2016). T cell receptor signalling in the control of regulatory T cell differentiation and function. Nature Reviews Immunology.

[ref-27] Lundback V, Kulyte A, Arner P, Strawbridge RJ, Dahlman I (2020). Genome-wide association study of diabetogenic adipose morphology in the GENetics of adipocyte lipolysis (GENiAL) cohort. Cells.

[ref-28] Malhab LJB, Saber-Ayad MM, Al-Hakm R, Nair VA, Paliogiannis P, Pintus G, Abdel-Rahman WM (2021). Chronic inflammation and cancer: the role of endothelial dysfunction and vascular inflammation. Current Pharmaceutical Design.

[ref-29] Marx S, Dal Maso T, Chen JW, Bury M, Wouters J, Michiels C, Le Calve B (2020). Transmembrane (TMEM) protein family members: Poorly characterized even if essential for the metastatic process. Seminars in Cancer Biology.

[ref-30] Moradi-Marjaneh R, Hassanian SM, Fiuji H, Soleimanpour S, Ferns GA, Avan A, Khazaei M (2018). Toll like receptor signaling pathway as a potential therapeutic target in colorectal cancer. Journal of Cellular Physiology.

[ref-31] Ness C, Katta K, Garred O, Kumar T, Olstad OK, Petrovski G, Moe MC, Noer A (2021). Integrated differential DNA methylation and gene expression of formalin-fixed paraffin-embedded uveal melanoma specimens identifies genes associated with early metastasis and poor prognosis. Experimental Eye Research.

[ref-32] Nie L, Zhang Y, You Y, Lin C, Li Q, Deng W, Ma J, Luo W, He H (2022). The signature based on seven genomic instability-related genes could predict the prognosis of acute myeloid leukemia patients. Hematology.

[ref-33] Qian Z, Zhang Z, Wang Y (2019). T cell receptor signaling pathway and cytokine-cytokine receptor interaction affect the rehabilitation process after respiratory syncytial virus infection. PeerJ.

[ref-34] Rahman R, Asombang AW, Ibdah JA (2014). Characteristics of gastric cancer in Asia. World Journal of Gastroenterology.

[ref-35] Reichman H, Karo-Atar D, Munitz A (2016). Emerging roles for eosinophils in the tumor microenvironment. Trends in Cancer.

[ref-36] Sammarco G, Varricchi G, Ferraro V, Ammendola M, De Fazio M, Altomare DF, Luposella M, Maltese L, Curro G, Marone G, Ranieri G, Memeo R (2019). Mast cells, angiogenesis and lymphangiogenesis in human gastric cancer. International Journal of Molecular Sciences.

[ref-37] Schmit K, Michiels C (2018). TMEM proteins in cancer: a review. Frontiers in Pharmacology.

[ref-38] Seidlitz T, Koo BK, Stange DE (2021). Gastric organoids-an in vitro model system for the study of gastric development and road to personalized medicine. Cell Death and Differentiation.

[ref-39] Shen K, Yu W, Yu Y, Liu X, Cui X (2018). Knockdown of TMEM45B inhibits cell proliferation and invasion in gastric cancer. Biomedicine & Pharmacotherapy.

[ref-40] Shiraishi T, Ikeda K, Tsukada Y, Nishizawa Y, Sasaki T, Ito M, Kojima M, Ishii G, Tsumura R, Saijou S, Koga Y, Yasunaga M, Matsumura Y (2021). High expression of TMEM180, a novel tumour marker, is associated with poor survival in stage III colorectal cancer. BMC Cancer.

[ref-41] Smyth EC, Nilsson M, Grabsch HI, van Grieken NC, Lordick F (2020). Gastric cancer. Lancet.

[ref-42] Song X, Jiang H, Qi Z, Shen X, Xue M, Hu J, Liu H, Zhou X, Tu J, Qi K (2020). APEC infection affects cytokine-cytokine receptor interaction and cell cycle pathways in chicken trachea. Research in Veterinary Science.

[ref-43] Takeda K, Akira S (2004). TLR signaling pathways. Seminars in Immunology.

[ref-44] Tan M, Schaffalitzky de Muckadell OB, Joergensen MT (2020). Gene expression network analysis of precursor lesions in familial pancreatic cancer. Journal of Pancreatic Cancer.

[ref-45] Tran Q, Park J, Lee H, Hong Y, Hong S, Park S, Kim SH (2017). TMEM39A and human diseases: a brief review. Toxicological Research.

[ref-46] Xin P, Xu X, Deng C, Liu S, Wang Y, Zhou X, Ma H, Wei D, Sun S (2020). The role of JAK/STAT signaling pathway and its inhibitors in diseases. International Immunopharmacology.

[ref-47] Xiong R, Yin T, Gao JL, Yuan YF (2020). HOXD9 activates the TGF-beta/Smad signaling pathway to promote gastric cancer. OncoTargets and Therapy.

[ref-48] Xu J, Su Z, Ding Q, Shen L, Nie X, Pan X, Yan A, Yan R, Zhou Y, Li L, Lu B (2019). Inhibition of proliferation by knockdown of transmembrane (TMEM) 168 in glioblastoma cells via suppression of Wnt/beta-catenin pathway. Oncology Research.

[ref-49] Zhang SH, Dai HT, Li WY, Wang RM, Wu HY, Shen M, Hu Y, Xie LX, Xing YM (2021a). TMEM116 is required for lung cancer cell motility and metastasis through PDK1 signaling pathway. Cell Death & Disease.

[ref-50] Zhang XL, He Y, Jiang Y, Bao Y, Chen QQ, Xie D, Yu HM, Wang X (2021b). TMEM229A suppresses non-small cell lung cancer progression via inactivating the ERK pathway. Oncology Reports.

[ref-51] Zhang X, Wang S, Wang HX, Cao JC, Huang XX, Chen Z, Xu PH, Sun GL, Xu JH, Lv JL, Xu ZK (2019). Circular RNA circNRIP1 acts as a microRNA-149-5p sponge to promote gastric cancer progression via the AKT1/mTOR pathway. Molecular Cancer.

[ref-52] Zhang L, Yu M, Deng J, Lv X, Liu J, Xiao Y, Yang W, Zhang Y, Li C (2015). Chemokine signaling pathway involved in CCL2 expression in patients with rheumatoid arthritis. Yonsei Medical Journal.

[ref-53] Zhang X, Zheng P, Li Z, Gao S, Liu G (2020). The somatic mutation landscape and RNA prognostic markers in stomach adenocarcinoma. OncoTargets and Therapy.

[ref-54] Zhao Y, Song K, Zhang Y, Xu H, Zhang X, Wang L, Fan C, Jiang G, Wang E (2018). TMEM17 promotes malignant progression of breast cancer via AKT/GSK3beta signaling. Cancer Management and Research.

[ref-55] Zhao Y, Zhang K, Pan H, Wang Y, Zhou X, Xiang Y, Xu Q, Sun Q, Tan J, Yan X, Li J, Guo J, Tang B, Liu Z (2022). Genetic analysis of six transmembrane protein family genes in Parkinson’s disease in a large Chinese cohort. Frontiers in Aging Neuroscience.

