# Supplementary material for: TMEM200A is a potential prognostic biomarker and correlated with immune infiltrates in gastric cancer
Source: PeerJ. 2023 Jun 29;11:e15613. doi: 10.7717/peerj.15613 (PMC10315132; doi:10.7717/peerj.15613)
Supplement: Supplemental Information 1 — (a) Cytokine-cytokine receptor interaction, (b) chemokine signaling pathway, (c) T cell receptor signaling pathway, (d) leukocyte transendothelial migration, (e) Toll-like receptor signaling pathway, (f) TGF-β signaling pathway, (g) JAK-STAT signaling pathway, (h) mTOR signaling pathway, (i) MAPK signaling pathway, (j) pathway in cancer. [file peerj-11-15613-s001.pdf]

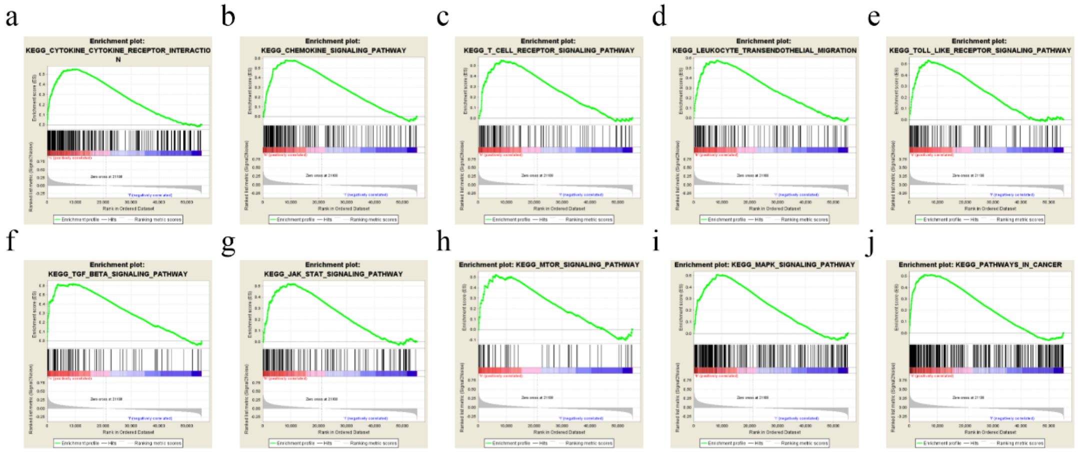

Figure S1: The significantly enriched signaling pathways associated with the increased TMEM200A expression. **(a)** Cytokine-cytokine receptor interaction, **(b)** chemokine signaling pathway, **(c)** T cell receptor signaling pathway, **(d)** leukocyte transendothelial migration, **(e)** Toll-like receptor signaling pathway, **(f)** TGF-β signaling pathway, **(g)** JAK-STAT signaling pathway, **(h)** mTOR signaling pathway, **(i)** MAPK signaling pathway, **(j)** pathway in cancer.
