# Supplementary material for: TMEM200A is a potential prognostic biomarker and correlated with immune infiltrates in gastric cancer
Source: PeerJ. 2023 Jun 29;11:e15613. doi: 10.7717/peerj.15613 (PMC10315132; doi:10.7717/peerj.15613)
Supplement: Supplemental Information 2 [file peerj-11-15613-s002.docx]

**Table S1:**

**Clinicopathological features of patients with GC.**

| **Clinicopathological features** | **classification** | **Patients (n=375)** | **Percentages (%)** |
| --- | --- | --- | --- |
| Age | <65 years | 155 | 41.33 |
|  | ≥65 years | 216 | 57.60 |
|  | Unknown | 4 | 1.07 |
| Gender | Male | 241 | 64.27 |
|  | Female | 134 | 35.73 |
| Grade | G1 | 10 | 2.67 |
|  | G2 | 137 | 36.53 |
|  | G3 | 219 | 58.40 |
|  | GX | 9 | 2.40 |
| Pathological stage | I | 53 | 14.13 |
|  | II | 111 | 29.60 |
|  | III | 150 | 40.00 |
|  | IV | 38 | 10.13 |
|  | Unknown | 23 | 6.14 |
| T | T1 | 19 | 5.07 |
|  | T2 | 80 | 21.33 |
|  | T3 | 168 | 44.80 |
|  | T4 | 100 | 26.67 |
|  | TX | 8 | 2.13 |
| N | N0 | 111 | 29.60 |
|  | N1 | 97 | 25.87 |
|  | N2 | 75 | 20.00 |
|  | N3 | 74 | 19.73 |
|  | NX | 16 | 4.27 |
|  | Unknown | 2 | 0.53 |
| M | M0 | 330 | 88.00 |
|  | M1 | 25 | 6.67 |
|  | MX | 20 | 5.33 |
| Vital status | Alive | 244 | 65.07 |
|  | Death | 131 | 34.93 |

Tumor, node, metastasis (TNM) classification.
