# Supplementary material for: TMEM200A is a potential prognostic biomarker and correlated with immune infiltrates in gastric cancer
Source: PeerJ. 2023 Jun 29;11:e15613. doi: 10.7717/peerj.15613 (PMC10315132; doi:10.7717/peerj.15613)
Supplement: Supplemental Information 3 [file peerj-11-15613-s003.docx]

**Table S2:**

**Relevant information of the selected GEO series dataset.**

| **GEO datasets** | **Platform** | **Sample** | **Total (*N*)** | **Country** | **Year** |
| --- | --- | --- | --- | --- | --- |
| GSE13195 | GPL5175 | GC | 25 | China | 2009 |
|  |  | Non-GC | 25 |  |  |
| GSE13911 | GPL570 | GC | 38 | Italy | 2008 |
|  |  | Non-GC | 31 |  |  |
| GSE26899 | GPL6947 | GC | 96 | USA | 2016 |
|  |  | Non-GC | 12 |  |  |
| GSE27342 | GPL5175 | GC | 80 | USA | 2011 |
|  |  | Non-GC | 80 |  |  |
| GSE33335 | GPL5175 | GC | 25 | China | 2012 |
|  |  | Non-GC | 25 |  |  |
| GSE54129 | GPL570 | GC | 111 | China | 2017 |
|  |  | Non-GC | 21 |  |  |
| GSE63089 | GPL5175 | GC | 45 | China | 2014 |
|  |  | Non-GC | 45 |  |  |
| GSE64591 | GPL570 | GC | 63 | USA | 2015 |
|  |  | Non-GC | 31 |  |  |
| GSE65801 | GPL14550 | GC | 32 | China | 2015 |
|  |  | Non-GC | 32 |  |  |
